# 

**Published:** 1891-09

**Authors:** 


### Sixteenth Quarterly Meeting of the Austin District Medical Society.

The Society will meet in Medical Hall, in this city, and will be called to order promptly at 9:30 a. m., Thursday, Sept. 24.

### PROGRAMME.

MORNING SESSION.

1. Reading of Minutes.
2. Application for Membership.
3. "Syphilis," by Dr. C. O. Weller; discussion opened by Drs. Matt M. Smith, and H. H. Thorpe.
4. "The Advantages of the Supra-Pubic Operation in Cystotomy," by Dr. W. M. Cunningham; discussion opened by Drs. W. J. Matthews and J. W. Hamilton.

AFTERNOON SESSION—2:30 O'CLOCK.

5. "Acne," by Dr. F. E. Daniel; discussion opened by Drs. Fannie Leake and L. H. Hardy.
6. "Biliousness," by Dr. W. E. Shelton; discussion opened by Drs. J. A. Davis and C. A. Danforth.
7. Voluntary papers.
8. Verbal Reports of Cases.
9. Unfinished Business.
10. New Business.

You are cordially invited to attend this meeting and participate in discussion of papers. We always have an interesting and profitable time. Study the subjects well and we will all be benefited. J. W. McLaughlin, President.

T. J. Bennett, Secretary.

